# Prevalence and associated factors of insomnia symptoms during the COVID-19 pandemic lockdown among Mettu town residents

**DOI:** 10.1371/journal.pone.0279624

**Published:** 2023-03-14

**Authors:** Mesfin Esayas Lelisho, Teramaj Wongel Wotale, Seid Ali Tareke

**Affiliations:** 1 Department of Statistics, College of Natural & Computational Science, Mizan-Tepi University, Tepi, Ethiopia; 2 Department of Statistics, College of Natural Science, Mettu University, Mettu, Oromia, Ethiopia; NYU Langone Health, UNITED STATES

## Abstract

**Background:**

Insomnia is a prevalent sleep disorder that affects people all over the world. Creating suitable interventions will require a better understanding of the magnitude and determinants of insomnia. This study aimed to assess the prevalence and associated factors of insomnia symptoms among residents of Mettu town during the pandemic lockdown.

**Methodology:**

A community-based cross-sectional study was conducted among residents of Mettu town from October 1^st^ to October 15^th^, 2020. Residents who lived in Mettu town at least for six months were included. To determine the prevalence and determinants of insomnia symptoms, both descriptive and inferential analyses were used. The chi-squared test of association and logistic regression was used to identify predictors of insomnia symptoms among residents of Mettu town. We used SPSS version 25 for all statistical analyses.

**Principal findings:**

The prevalence of depressive symptoms among residents of Mettu town was 52.6%. According to results of multivariable binary logistic regression, being female [AOR = 3.677, 95%CI: 2.124–6.365], being aged between 19 and 40 [AOR = 13.261, 95%CI: 6.953–25.291], being aged above 41 [AOR = 2.627, 95%CI: 1.120–6.159], smoking [AOR = 15.539, 95%CI: 7.961–30.329], satisfaction with information available [AOR = 0.310, 95%CI: 0.168–0.570], fear Corona Virus Disease 2019 (COVID-19), [AOR = 2.171, 95%CI: 1.262–3.733], feeling alienated from others [AOR = 3.288, 95%CI: 1.897–5.699], having somatic symptoms [AOR = 2.298, 95% CI: 1.360–3.884], having depressive symptoms [AOR = 1.841, 95% CI: 1.073–3.160], and experiencing psychological distress [AOR = 1.962, 95% CI: 1.173–3.281] were significantly associated with insomnia symptoms.

**Conclusion:**

In this study, the prevalence of insomnia symptoms was found to be high among residents of Mettu town. Being female, being aged between 19 and 40, being aged above 41 years, smoking, fear of Corona Virus Disease 2019, feeling alienated from others, having somatic symptoms, having depressive symptoms, and experiencing psychological distress were all associated with an increased risk of developing insomnia symptoms while being satisfied with the information available decreased the risk of insomnia symptoms among residents of Mettu town. Interventions should be put in place to promote healthy sleep among residents of Mettu town.

## Introduction

Coronavirus disease was first reported in December 2019 in Wuhan, China, and is fast spreading all over the world since then. On thirty January 2020, the World Health Organization declared it to be a global public health disaster, and on 11 March 2020, a pandemic [1]. During the COVID-19 pandemic, relatively high rates of anxiety, depression, post-traumatic stress disorder, psychological distress, and stress were reported in the general population in China, Spain, Italy, Iran, the United States, Turkey, Nepal, and Denmark [2]. The influence of COVID-19 and its link with physical health among three countries was reported by Wang et al., who found that Poland and the Philippines had the highest levels of anxiety, depression, and stress, while Vietnam had the lowest mean scores in these categories [3]. COVID-19 has had a considerable impact on developing countries [4–7]. A comparative study of a multi-national survey conducted across 7 MICs in Asia showed that Thai-wan had the highest mean IES-R and anxiety, depression, and stress ratings [8].

During the COVID-19 pandemic, countries throughout the world took a variety of strategic measures to combat the virus. They have implemented containment measures such as contact limitations, self-isolation, school and college/university closures, quarantine, and social distancing [9]. Following the implementation of social distancing due to the impact of COVID-19, the rate of household income loss as well as degradation of various quality of life dimensions among the general population in Vietnam have increased [10]. In China, more than half of respondents ranked the psychological impact of the COVID-19 outbreak as moderate-to-severe, and nearly a third expressed moderate-to-severe worry during the early stages of the outbreak [11]. While the long-term psychological repercussions of the lockdown are still unknown, early symptoms of worry, tension, and sadness among people can be used as a predictor of the long-term psychological impact [12].

Mental health professionals believe that the epidemic and the resulting lockdown will influence the mental health of the global population, with an increase in cases of insomnia, sadness, and self-harm [13]. During the outbreak, insomnia, sadness, and acute stress symptoms were all shown to be more prevalent in the general population [14, 15]. Following COVID, 3–12% of people had clinically significant depression and/or severe depressive symptoms [16]. In contrast, psychiatric patients had higher levels of anxiety, depression, stress, and insomnia than the general population during the COVID-19 outbreak as a result of strict lockdown measures [17]. On the other hand, in terms of impulsivity and insomnia, COVID-19 patients had higher levels of neuropsychiatric symptoms than psychiatric patients without COVID-19 and healthy individuals in the community. They experienced emotions such as shock, fear, boredom, and hope during their treatment, as well as common concerns about discrimination, medical expenses, healthcare workers’ care, and self-help behavior [18].

Insomnia is a serious health problem with symptoms such as difficulty falling asleep, staying asleep, and waking up early in the morning, and it has become a major public health issue around the world [19, 20]. It is also linked to daytime exhaustion, decreased activity, absenteeism, low quality of life, and major medical and societal expenditures [21, 22]. Previous studies have found that insomnia can induce diseases such as hypertension, diabetes, and cardiovascular disease [23]. It also contributes to psychological disorders such as anxiety, sadness, bipolar disorder, and suicidal ideation [24, 25]. Because insomnia has serious effects, including depression, poor work performance, and overall poor quality of life [26], examining the prevalence and associated characteristics of insomnia is critical for developing prevention, interventions, and allocating health resources. However, research on sleep problems like insomnia in developing countries including Ethiopia is scarce. Therefore, the primary goal of the study was to measure the prevalence of insomnia in the Mettu town population. Our secondary interest was identifying factors associated with insomnia among Mettu town residents, in southwest Ethiopia.

## Materials and methods

### Study design and setting

From October 1^st^ to October 15^th^, 2020, a community-based cross-sectional study was conducted on residents of Mettu Town in Southwestern Ethiopia. A self-administered questionnaire was used to collect data from town residents chosen using a simple random sampling technique. Residents must have lived in Mettu for at least six months to be included in our sample. Participants who were unable to provide information were excluded from the study.

#### Sampling procedure

The sample size was calculated using a single population proportion formula, and a simple random sampling technique. The sample size needed for the study was calculated by assuming 50% of the prevalence of insomnia among residents in a single population fraction for unknown prevalence, with a margin of error of 4%, and a non-response rate of 10%. Then the sample size for this study becomes 600.25+60≈661.

#### Study variables

The response variable was insomnia symptoms (*yi*), which is dichotomized as

$$yi=\left\{ \begin{array}{l} 1,\;presence\;of\;insomnia\;symptoms\\ 0,\;otherwise \end{array}\right.$$

Explanatory variables:

Demographic variables (gender, age (in years), education level, alcohol consumption, chewing Khat, smoking status). COVID-19-related perception (COVID-19 infection, fear of COVID-19, anyone around with confirmed or suspected of COVID-19 infection, the satisfaction of available information, feeling alienated from others), Psychological factors (depressive symptoms, somatic symptoms, and psychological distress)

#### Data collection tools and measurements

To determine the symptoms of insomnia, the Insomnia Severity Index (ISI) was used. It is a reliable and valid instrument to quantify the severity of perceived insomnia [27]. A score greater than or equal to eight indicates the presence of insomnia symptoms [28, 29].

To examine depressive symptoms, the Patient Health Questionnaire-9 (PHQ-9) was used [30]. There are five severity levels to which we categorize the total score: normal (0–4), mild depression (5–9), moderate depression (10–14), moderate to severe depression (15–19), and severe depression (20–27) [31, 32]. The reliability and validity of the PHQ-9 had proved great among the Chinese general population [32]. The sum score ≥ 5 on PHQ-9 is assumed to have depressive symptoms [31].

For the evaluation of psychological distress in response to COVID-19, the Impact of Events Scale-Revised (IES-R) was performed [33]. The total scores can be categorized into four different levels on a five-point Likert scale: subclinical (0–8), mild (9–25), moderate (26–43), and severe (44–88). In the Chinese version of the IES-R, high reliability and validity were identified. The sum score ≥ 9 on IES-R is assumed to have psychological distress symptoms [34].

To evaluate the somatic symptom, we used the Somatic Symptom Scale–8 (SSS-8), which is composed of eight items and used to evaluate the somatic symptom burden over the past 7 days. The total sum of the scores can be ranged from 0–32 which can be categorized into five different levels: none to minimal (0–3), low (4–7), medium (8–11), high (12–15), or very high (16–32) physical symptoms [35] Matsudaira’s study confirmed the validity and internal consistency of SSS-8. The sum score ≥ 4 on SSS-8 is assumed to have somatic symptoms [36].

#### Method of data analysis

To highlight descriptive results, we used frequency distribution and percentages. The chi-squared test of association was employed to examine the relationship between response and explanatory variables. The logistic regression model was utilized to find determinants of insomnia symptoms. Multivariable logistic regressions were conducted by taking all significant covariates in the Univariable analysis at a significance level of 25% [37]. The Hosmer and Lemeshow test is used for determining the model’s goodness of fit. In the current study, SPSS version 25 was used for all statistical analyses [38].

*Binary logistic regression*. When the dependent variable is dichotomous, such as the presence or absence of a specific event, and the independent variables are of any type, binary logistic regression is used. The Bernoulli distribution for the Bernoulli trial specifies probabilities P (Y = 1) = π and P(Y = 0) = 1- π, for which E (Y) = π.

The general model for binary logistic regression is as follows:

$$\log it(\pi (xi))=\log(\frac{\pi (xi)}{1-\pi (xi)})=\beta o+\beta_{1}X_{1}+\beta_{2}X_{2}+....+\beta_{K}X_{k}$$ (1)

Where: *x_i_* is an independent variable in the model, π: the probability of success, 1-π: the probability of failure, *β_o_* is constant terms, *β_i_* and is the coefficients/slope of the independent variable in the model.

*Ethical clearance and consent to the publication*. Ethical clearance was obtained from the research review committee of the College of Natural Science, Mettu University. The objective of the study was explained to the participants. Since the participants included all of the town’s societies, including those who were illiterate and hesitant to give signatures or thumbprints on consent forms, verbal informed consent was obtained from all participants. In the event of minors, the home leader (father, mother, or guardians) gave oral consent first, followed by all child participants. Participants were also informed to have the right not to participate in the study if they were not interested to be involved in it.

## Results

**Fig 1:**
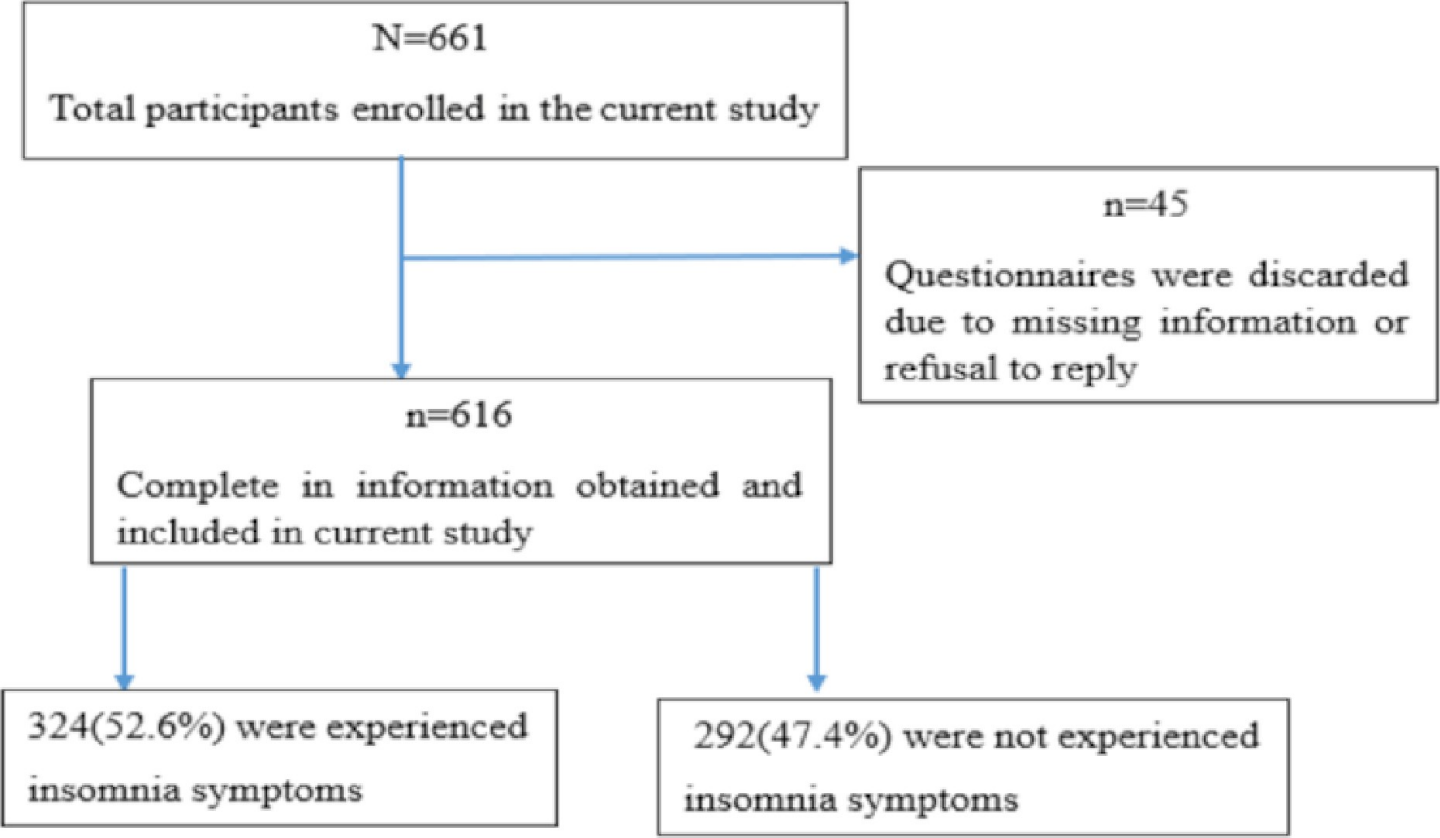
Illustrative summary of major findings.

This study was carried out to assess the prevalence and predictors of insomnia symptoms among the residents of Mettu Town, southwest Ethiopia, during the COVID-19 pandemic lockdown. In this study, both descriptive and inferential analyses have been used to assess the prevalence and predictors of insomnia symptoms. The sample size of this study was 661, however, 45 questionnaires were discarded due to missing information or refusal to reply, resulting in a total of 616 participants. Of 616 study participants, 324(52.6%) experienced insomnia symptoms, while 292(47.4%) did not (**Fig 1**).

### Demographic characteristics of the participants

The majority of the 616 participants in this study 354(57.5%) were males, with 164(46.3%) developing insomnia symptoms, while females were 262(42.5%), with 160(61.1%) reporting/experiencing insomnia symptoms. More than half of the responders 345(56.0%) were between the ages of 19 and 40, with 258 (74.8%) experiencing insomnia symptoms. In terms of education, 84 (13.6%) of respondents had no formal education, 356 (57.8%) had elementary or secondary school, and 176 (28.6%) had college or higher education (Table 1).

**Table 1:** Demographic profile of respondents and insomnia symptoms.

Variable	Categories	Insomnia symptoms		Total N (%)	P-value*
		Yes, N (%)	No, N (%)		
Gender	Male	164(46.3)	190(53.7)	354(57.5)	< .001
	Female	160(61.1)	102(38.9)	262(42.5)	
Age (in years)	≤ 18 years	33(17.6)	155(82.4)	188(30.5)	< .001
	19–40	258(74.8)	87(25.2)	345(56.0)	
	≥41	33(39.8)	50(60.2)	83(13.5)	
Education status	No formal education	53(63.1)	31(36.9)	84(13.6)	0.36
	Primary/Secondary	173(48.6)	183(51.4)	356(57.8)	
	College and above	164(46.3)	78(44.3)	176(28.6)	
Alcohol consumption	No	189(47.7)	207(52.3)	396(64.3)	< .001
	Yes	135(61.4)	85(38.6)	220(35.7)	
Chewing Khat	No	172(53.1)	152(46.9)	324(52.6)	0.43
	Yes	152(52.1)	140(47.9)	292(47.4)	
Smoking status	No	154(36.9)	263(63.1)	417(67.7)	< .001
	Yes	170(85.4)	29(14.6)	199(32.3)	

*Pearson chi-square p-value.

In terms of substance usage, 220 (35.7%), 292 (47.3%), and 199 (32.3%) of respondents were alcohol users, Khat chewers, and smokers, respectively, with 61.4%, 52.1%, and 85.4% experiencing insomnia symptoms (Table 1).

### COVID-19-related perception and insomnia symptoms

Out of the total, 277 (45.0%) reported concern about COVID-19 infection, with 206 (74.4%) experiencing insomnia symptoms. In response to the question "if someone around with confirmed COVID-19 infection," 47 (7.6%) reported having a family member infected with COVID-19, with more than one-third (38.3%) developing insomnia symptoms. It is also found that about two-thirds of respondents (60.7%) were dissatisfied with the available information, and approximately half of 295 (47.9%) felt alienated from others (Table 2).

**Table 2:** COVID-19-related perception and insomnia symptoms.

Variables	Categories	Insomnia symptom			P-value*
		Yes, N (%)	No, N (%)	Total, N (%)	
COVID-19 infection	No	216(51.8)	201(48.2)	417(67.7)	.065
	Suspected	74(56.1)	58(43.9)	132(21.4)	
	Confirmed	34(50.7)	33(49.3)	67(10.9)	
Fear of COVID-19 infected	No	118(34.8)	221(65.2)	339(55.0)	< .001
	Yes	206(74.4)	71(25.6)	277(45.0)	
Anyone around with confirmed COVID-19 infection	None	228(54.0)	194(46.0)	422(68.5)	.013
	Family	18(38.3)	29(61.7)	47(7.6)	
	Friend	60(56.1)	47(43.9)	107(17.4)	
	Neighbor	18(45.0)	22(55.0)	40(6.5)	
Satisfaction of available information	Dissatisfied	180(48.1)	194(51.9)	374(60.7)	.004
	Satisfied	144(59.5)	98(40.5)	242(39.3)	
Feeling alienated from others	No	95(29.6)	226(70.4)	321(52.1)	< .001
	Yes	229(77.6)	66(22.4)	295(47.9)	
Depressive symptoms	No	166(43.2)	218(56.8)	384(62.3)	< .001
	Yes	158(68.1)	74(31.9)	232(37.7)	
Somatic symptoms	No	137(38.7)	217(61.3)	354(57.5)	< .001
	Yes	187(71.4)	75(28.6)	262(42.5)	
Psychological distress	No	114(39.6)	174(60.4)	288(46.8)	< .001
	Yes	210(64.0)	118(36.0)	328(53.2)	

*Pearson chi-square p-value.

The findings in (Table 2) show psychological characteristics and insomnia symptoms among study participants. Depressive symptoms, somatic symptoms, and psychological distress were present in approximately 232 (37.7 percent), 262 (42.5 percent), and 328 (53.2 percent) of the population, respectively. Insomnia symptoms were experienced by 68.1 percent, 71.4 percent, and 64.0 percent of those polled.

### Univariable analysis

In the Univariable analysis, covariates with a p-value less than 25% were considered for multivariable analysis. From the Univariable analysis, we observed that the covariate gender, age (in years), alcohol drinking habit, smoking habit, anyone around with confirmed COVID-19 infection, the satisfaction of available information, feeling alienated, depressive symptoms, somatic symptoms, and psychological distress were significant. However, chewing Khat and education level was not a significant at 25% level of significance. Therefore, based on this result, it is better to ignore this covariate and shall do our multivariable analysis using the significant factors. Hence, the effects of these significant covariates shall better be interpreted using the multivariable analysis.

### Multivariable analysis

Gender, age, smoking habit, satisfaction with available information, fear of COVID-19, feeling alienated, anyone around infected with COVID-19, somatic symptoms, depressive symptoms, and psychological distress were all found to be statistically significant at the 5% level of significance in a multivariable binary logistic regression (Table 3).

**Table 3:** Multivariable binary logistic regression result for insomnia among Mettu town residents, southwest Ethiopia.

Variables	Categories	B	S.E.	Sig.	EXP(B)	95%C.I.for EXP(B)	
						Lower	Upper
Gender (ref: Male)	Female	1.302	.280	.000	3.677	2.124	6.365
Age (in years) (ref: ≤ 18 years)	19–40	2.585	.329	.000	13.261	6.953	25.291
	≥ 41	.966	.435	.026	2.627	1.120	6.159
Alcohol consumption (ref: No)		-.209	.276	.449	.812	.473	1.393
Smoking habit (ref: No)		2.743	.341	.000	15.539	7.961	30.329
COVID-19 infection (ref: None)	Suspected	.041	.318	.897	1.042	.558	1.944
	Confirmed	.001	.402	.999	1.001	.455	2.199
Satisfaction with information (ref: No)		-1.171	.311	< .001	0.310	0.168	0.570
Fear of COVID-19 (ref: No)		.775	.277	.005	2.171	1.262	3.733
Feeling Alienated (ref: No)		1.190	.281	.000	3.288	1.897	5.699
Anyone infected with COVID-19 (ref: None)	Families	.984	.327	.002	2.675	1.409	5.078
	Friends or colleagues	.284	.430	.510	1.328	0.572	3.086
	Neighborhood	1.430	.869	.052	0.239	0.761	22.95
Somatic symptoms (ref: No)		.832	.268	.002	2.298	1.360	3.884
Depressive symptoms (ref: No)		.610	.276	.027	1.841	1.073	3.160
Psychological distress (ref: No)		.674	.262	.010	1.962	1.173	3.281
Nagelkerke’s R Square	0.688						
Hosmer and Lemeshow Test	0.486						

B: Coefficient, S.E.: Standard error, Sig.: p-value, EXP (B): Adjusted Odds Ratio, C.I.: 95% confidence interval for adjusted odds ratio, ref: reference category.

Females were 3.677 [95% CI: 2.124–6.365] times more likely than males to have a prevalence of insomnia symptoms. Insomnia symptoms were substantially connected with increasing age. Respondents between the ages of 19 and 40 were 13.261 [95% CI: 6.953–25.291] times more likely to have insomnia symptoms, while those over 41 years old were 2.627 [95% CI: 1.120–6.159] times more likely to have insomnia symptoms than those under the age of 18. When compared to non-smokers, smokers were 15.539 [95% CI: 7.961–30.329] times more likely to develop insomnia symptoms.

Another element connected with insomnia symptoms was satisfaction with available information. Those who were satisfied with the provided information were 0.310 [95% CI: 0.168–0.570] times less likely to suffer insomnia symptoms than those who were dissatisfied. Respondents who were afraid of COVID-19 had a two-fold (2.171) [95% CI: 1.262–3.733] greater chance of suffering insomnia symptoms than those who were not afraid of COVID-19. In comparison to individuals who did not feel alienated, those who felt alienated were 3.288 [95% CI: 1.897–5.699] times more likely to have insomnia symptoms. Respondents who had family members infected with COVID-19 were 2.675 [95% CI: 1.409–5.078] times more likely to have insomnia symptoms than those who did not have any suspected or infected COVID-19 infected persons around them.

In addition, participants with depressive symptoms 1.841 [95% CI: 1.073–3.160], somatic symptoms 2.298 [95% CI: 1.360–3.884], and psychological distress 1.962 [95% CI: 1.173–3.281] had an increased risk of insomnia symptoms when compared to their respective reference group (Table 3).

#### Model adequacy checking

The Hosmer and Lemeshow test result is large (p-value = 0.486), indicating that the model was a good fit for the data. Furthermore, Nagelkerke’s R square (0.688) revealed that existing explanatory variables in the model explained 68.8% of the variation among response variables, while error terms and unknown factors accounted for the remaining 31.2% (Table 3).

**Fig 2:**
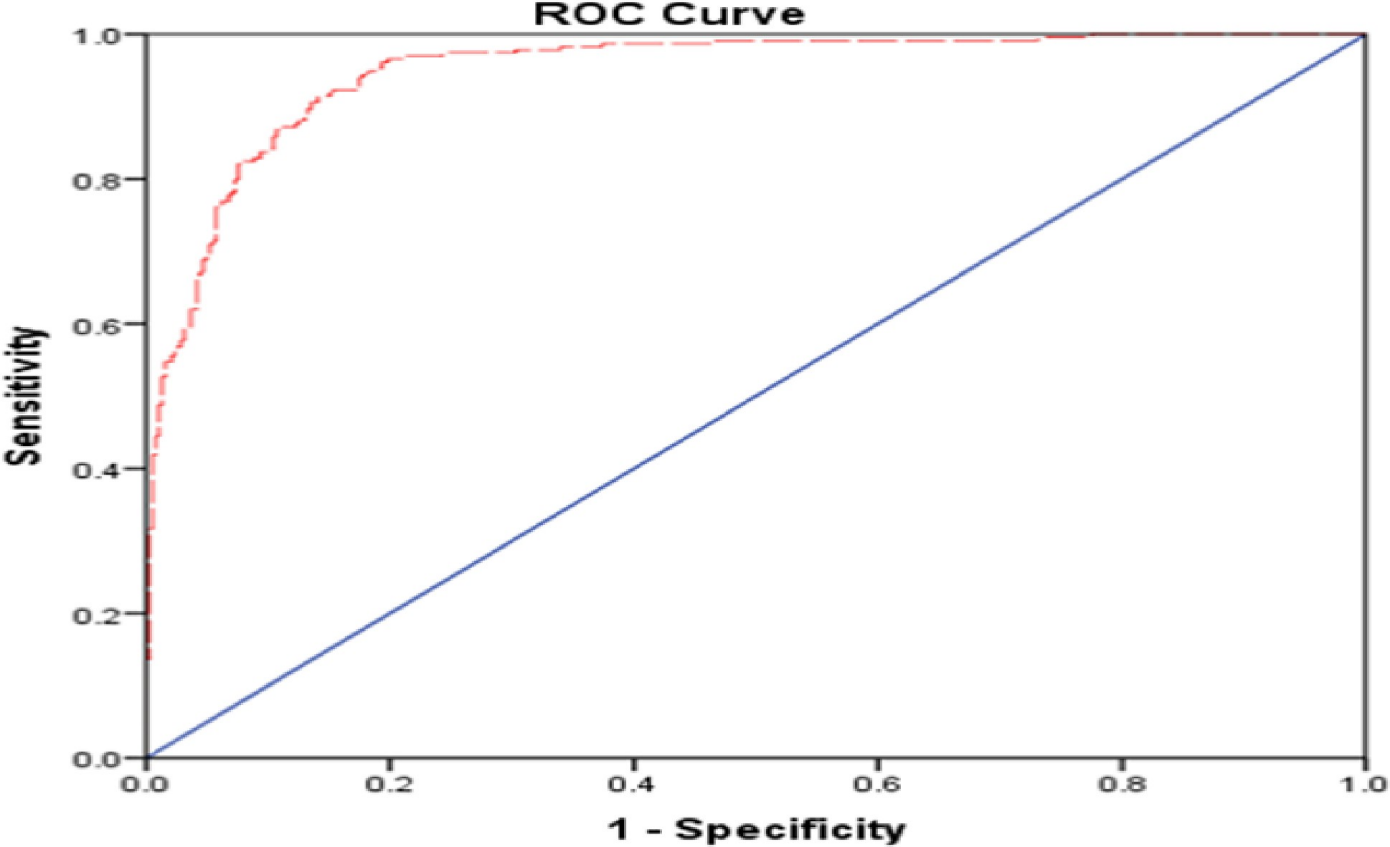
Receiver operating characteristics curve for the fitted model.

The Reciever operating characteristics curve (ROC curve) plots the probability of detecting true signal (sensitivity) and false signal (1-specificity) for an entire range of possible cut points. The area under the ROC curve indicates how effective the test is in a numerical sense. In the case of this study, for the fitted model (Table 1), a plot of sensitivity versus 1-specificity over all possible cut points is shown (**Fig 2**). The model performance is regarded as excellent if the area is 0.8≤ROC≤0.9, while more than 0.9 is considered outstanding. Our result showed that the area under this curve is determined by the Mann-Whitney U statistic and is 0.879. Therefore, based on the area under the curve indicates our model performance is excellent to predict the event.

**Fig 3:**
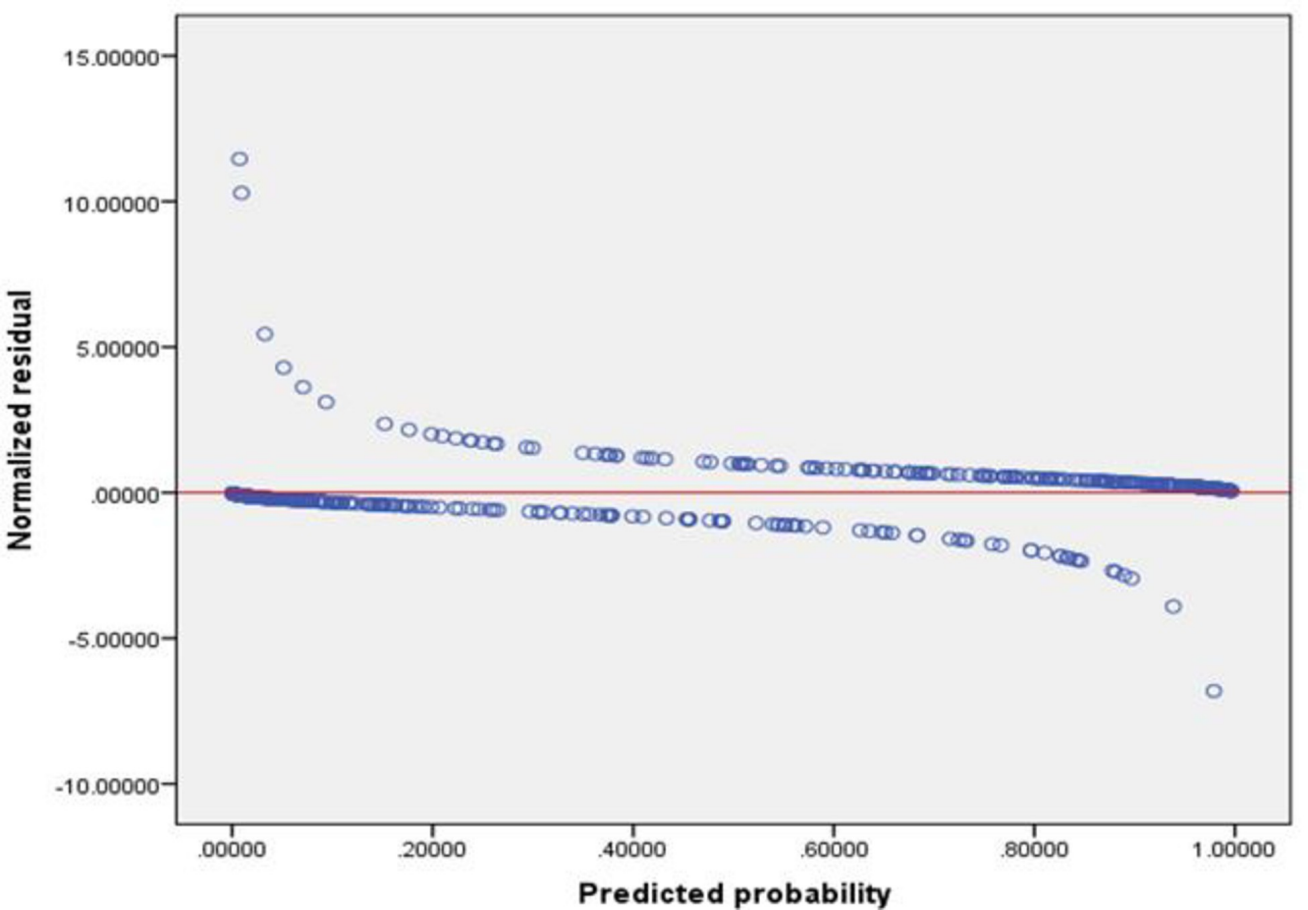
Residual versus predicted probability plot for the fitted model.

Furthermore, if the model is a good fit, then the absolute values of the residuals are relatively small, and the residual points will be more or less evenly dispersed about the horizontal axis. In the current study, residual values dispersed around the horizontal reference line, indicate that the model is a good fit for the data (**Fig 3**).

## Discussion

The purpose of this study was to determine the prevalence and associated factors of insomnia symptoms among Mettu town residents during the pandemic lockdown. Understanding the factors associated with insomnia symptoms could help to provide precise interventions for insomnia in the public. Moreover, it contributes to the evidence of the effects of the COVID-19 pandemic on mental health. Besides, this is one of the few studies in developing countries that used standardized measurement tools and conducts rigorous analyses. In the current study, being female, being older, having smoking habits, satisfaction with the information available, fear of COVID-19, feeling alienated from others, having somatic symptoms, having depressive symptoms, and experiencing psychological distress were all found to be risk factors for insomnia symptoms.

Insomnia affects 10%– 30% of the population worldwide, with some estimates reaching 50%–60% [26]. In the present study, the prevalence of insomnia symptoms was 52.6 percent. This is consistent with a previous report from India at 53.45% [39], and Turkey at 51.0% [40]. However, the current studies report was higher than previous reports, China’s general public, 30.9% [41], Saud Arabia 34.9% [42], Greek 37.6% [43], Dhaka, Bangladesh among healthcare workers 44.2% [44], systematic review and meta-analysis of 13 countries 36.0% [45], Greek 37.6% [43], and Ethiopia 42.9% [46]. Another study from the United States shows that the prevalence of insomnia symptoms in adolescents ranges between 3.4 and 34.6% [47, 48]. Similarly in a group of school teachers in Portugal, 40.6% reported experiencing sleeplessness symptoms [49]. This difference might be due to different study designs, populations, and cultural differences. While some research found an even higher prevalence of Insomnia symptoms, multi-country reports among recovered COVID-19 patients revealed a prevalence of 77.6 percent [50]. This increased prevalence of insomnia symptoms could be attributed to the fact that they only included COVID-19 infected and recovered patients.

### Significant factors associated with insomnia symptoms

Our study findings revealed that gender was found to be a significant predictor of insomnia symptoms among study participants. Females were more likely than males to experience insomnia symptoms. Females may be burdened by familial duties like many tasks and responsibilities, gender discrimination such as gender-based violence, and concomitant common mental illnesses such as depression and anxiety, which are more common in females than in males [51]. Previous studies [52, 53] reported the same result.

According to the findings of the current study, age has a strong relationship with insomnia symptoms. Insomnia symptoms were more common in older people than in younger people. In our study, people between the ages of 19 and 40 years, as well as those above 41 years had greater insomnia symptoms than those under the age of 18. This is consistent with earlier research, which found that the rate of insomnia symptoms increased with age and that insomnia symptoms were more likely to occur in the elderly [54–56]. The physiological changes in sleep and circadian rhythm that occur during life might explain this phenomenon [57, 58]. Additionally, stressful life events or medical problems, such as respiratory difficulties, physical impairment, and poor perceived health, enhance the incidence of sleeplessness in older individuals [58].

In our study, smokers had a higher incidence of insomnia symptoms than nonsmokers, which is consistent with previous findings [59, 60]. Numerous studies have revealed that smokers are more likely to experience the symptoms of insomnia, including leg movements while sleeping more frequently, shorter sleep duration, and higher rapid eye movement [61–63]. Accordingly, numerous earlier studies have demonstrated a connection between smoking, especially late-night smoking, and more severe insomnia and shorter sleep duration [61–63]. According to research done by Sabanayagam & Shankar (2011), current smokers of cigarettes were almost twice as likely to report not getting enough rest and sleep as opposed to non-smokers [64]. Furthermore, Andrea, et al. (2021) suggested that quitting smoking could reverse the detrimental effects of smoking on sleep [65]. As a result, sleep health should be promoted in programs to help smokers quit to reduce their chances of developing insomnia.

In line with a previous study [39], the current study revealed that people who fear COVID-19 have greater sleeplessness than those who are unconcerned. This finding is consistent with prior evidence on insomnia and COVID-19-related concerns [66]. One of the most powerful predictors of sleep disruption is worry [67]. Worrying thoughts, such as repeated thoughts, negative overthinking, cognitive arousal, and intrusive thoughts, have been linked to insomnia symptoms [68]. Worrying about unpleasant situations can also contribute to hyper-arousal, which is a major cause of insomnia [69].

Respondents’ feelings of alienation were revealed to be a risk factor for the development of insomnia symptoms. According to a prior study, more acute insomnia at the baseline was associated with worse parental connections and more peer issues [70]. In line with this, in Greece, those who felt alienated were more likely to suffer from insomnia symptoms [43]. In COVID-19 isolation, Matias et al. (2020) describe human needs and suggest that protective practices that are good for one’s health be encouraged [71]. In support of this, Ana Veronica Scott et al. studied participants’ physical activity and analyzed its relationship with insomnia, finding that physically active participants had lower ISI scores [72]. So, to alleviate the loneliness that causes insomnia, we suggested physical activities.

A prior study found that those with confirmed or suspected family members, friends, and residents had more severe symptoms of mental health issues [73]. In line with this report, the current study revealed that the existence of a family member infected with COVID-19 was significantly associated with insomnia symptoms. This is also confirmed by a recent study [74, 75], which discovered that persons having family members infected with COVID-19 had a higher risk of having sleeplessness issues. Another study discovered that one out of every five survivors’ family members was diagnosed with a mental disease for the first time, including sleeplessness symptoms [76–78].

Satisfaction with available information was discovered to be another factor associated with insomnia symptoms. In the current study, those who were satisfied with the information provided were less likely to experience insomnia symptoms than those who were unsatisfied. This is in agreement with a previous study from China [56]. Obtaining appropriate knowledge about the virus from social and mass media may assist them in avoiding stress, and sleeplessness symptoms. Previous research [56, 79] found a link between depressive symptoms, somatic symptoms, psychological distress, and the onset of insomnia symptoms [80]. In line with prior research, the current study found that depressive symptoms, somatic symptoms, and psychological distress were all substantially related to insomnia symptoms.

Various intervention strategies were used during the COVID-19 outbreak to assist patients with treating mental health issues, including insomnia. CBT (cognitive behavioral therapy) is a versatile strategy for treating a variety of mental illnesses [81]. During an outbreak, several institutions have shifted to providing online psychotherapy to those suffering from mental health disorders via video conferencing platforms to reduce viral transmission from face-to-face therapy. Furthermore, providing online or smartphone-based psychoeducation regarding the virus’s spread, promoting mental well-being, and initiating psychological intervention might be beneficial (e.g. cognitive behavior therapy [CBT] and mindfulness-based therapy [MBT]) [82]. In addition, digital cognitive behavioral therapy for insomnia (dCBT-I) [83], and Internet CBT (iCBT) [84] are effective therapeutic options for patients with insomnia. The effectiveness of dCBT-I in treating insomnia is supported by a meta-analysis of randomized controlled trials, and dCBT-I has the potential to revolutionize CBT-I delivery by increasing the accessibility and availability of CBT-I information for insomnia patients around the world [83].

### Limitations of the study

The current study tried to assess insomnia symptoms among residents of Mettu towns, in southwest Ethiopia. There are some limitations while conducting this study. Firstly, we cannot prove a causal relationship in this cross-sectional study. As a second point, a self-reported questionnaire was conducted, which will contribute to a certain amount of answer bias. Finally, in addition to the variables we considered, there may be other factors related to the prevalence of insomnia symptoms among residents that can cause insomnia, which requires further investigation.

## Conclusions

The current study found that residents had experienced a higher prevalence of insomnia symptoms during the COVID- 19 pandemic. Being female, being older, smoking, having fear of COVID-19, feeling alienated from others, having somatic symptoms, having depressive symptoms, and experiencing psychological distress were all associated with an increased risk of developing insomnia symptoms, while satisfaction with information available decreased the risk of insomnia symptoms among Mettu town residents. Measures to enhance the mental health of people who had sleeplessness symptoms should be done based on significant factors and responsible bodies should endeavor to safeguard them. Interventions based on influencing factors should be implemented to ensure the sleep quality of residents.

## Supporting information

S1 Data (CSV)

## List of abbreviations

COVID-19: Corona Virus Disease 2019
PHQ-9: Patient Health Questionnaire-9
SSS-8: Somatic Symptom Scale–8
IES-R: Impact of Events Scale-Revised
U.S.: United States
